# Varicocele due to renal arteriovenous malformation mimicking a renal tumor: a case report

**DOI:** 10.1186/s13256-017-1546-2

**Published:** 2018-01-05

**Authors:** Peng-chao Li, Jia-yi Zhang, Yan-yan Xiu, Sheng Liu, Jin-guo Xia, Hai-bin Shi, Ning-hong Song

**Affiliations:** 1 Department of Urology, The First Affiliated Hospital of Nanjing Medical University, Nanjing, China; 2 Department of Dermatology, The First Affiliated Hospital of Nanjing Medical University, Nanjing, China; 3 Department of Radiology, The First Affiliated Hospital of Nanjing Medical University, Nanjing, China

**Keywords:** Varicocele, Arteriovenous malformations, Digital subtraction angiography

## Abstract

**Background:**

Renal arteriovenous malformation is an aberrant vascular connection between the renal artery and vein. Acquired renal arteriovenous malformation (arteriovenous fistulae) accounts for approximately 70% of renal arteriovenous abnormalities. Congenital renal arteriovenous malformation, relatively rare, can result in significant hematuria which may require arterial embolization or nephrectomy.

**Case presentation:**

A 64-year-old Asian man presented to the Urology department in our hospital with gradual left scrotal swelling for 2 years. Ultrasound and computed tomography showed an irregular mass in the upper pole of his left kidney. Digital subtraction angiography confirmed cirsoid-type left renal arteriovenous malformation combined with left renal vein ostial stenosis. After digital subtraction angiography and selective segmental renal artery embolization, the varicocele was obviously alleviated.

**Conclusions:**

The etiology diagnosis of varicocele is not always straightforward, and renal arteriovenous malformation should be considered in the differential diagnosis of varicocele and renal mass. Renal arteriovenous malformation is difficult to distinguish from renal tumor according to varicocele and computed tomography presentation, while magnetic resonance imaging and digital subtraction angiography help to make a definite diagnosis and selective renal angiographic embolization is one of the best treatments for renal arteriovenous malformation.

## Background

Renal arteriovenous malformation (AVM) is an aberrant vascular connection between the renal artery and vein. AVM can be either acquired or congenital. Acquired renal AVM (arteriovenous fistulae, AVF) accounts for approximately 70% of renal arteriovenous abnormalities and usually results from trauma, biopsy, surgery, or inflammation. Congenital renal AVM, relatively rare, can result in significant hematuria which may require arterial embolization or nephrectomy [1]. Other presentations including hypertension of unknown cause, abdominal pain, cardiac failure, and left ventricular hypertrophy are also usually associated with AVM [2]. Varicocele is present in 15% of adult men, and is a rare symptom of renal cell carcinomas (RCCs) with inferior vena cava tumor thrombus [3, 4].

We report a case that presented as severe varicocele, and suspicious malignant renal mass was suggested by computed tomography (CT). The case did not show the typical symptoms of renal AVM including hematuria, hypertension, left ventricular hypertrophy, cardiac failure, abdominal pain, or flank pain, which increased the difficulty of diagnosis. This patient was finally diagnosed as having renal AVM combined with renal vein ostial stenosis after magnetic resonance imaging (MRI) and selective renal arteriography were conducted. Therefore, varicocele could be a suspicious presentation for asymptomatic renal AVM, which requires the confirmation of renal arteriography.

## Case presentation

**Fig. 1:**
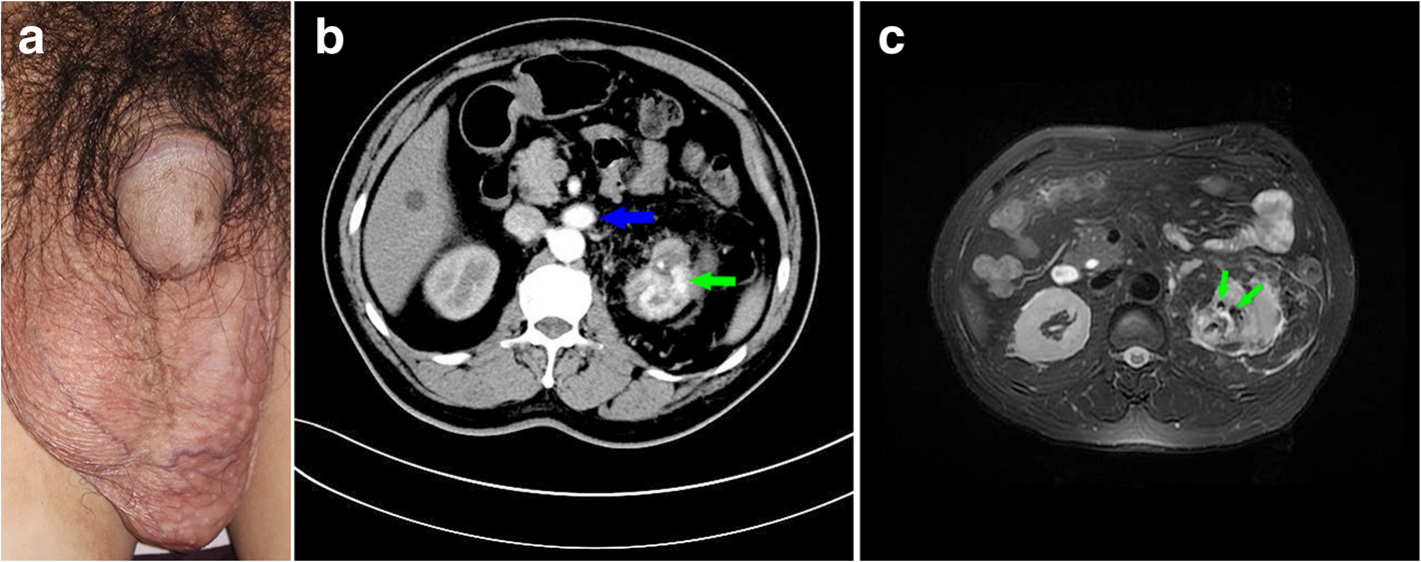
**a** Physical examination showed grade 3 left varicocele. **b** Computed tomography showed early enhanced dilated renal vein (*blue arrow*) and irregular lesion in the upper pole of left kidney, which consisted of unusual dilated enhanced vessel (*green arrow*) in renal cortical phase. **c** T2 magnetic resonance imaging showed abnormal early flow voids (*green arrows*) in the upper pole of left kidney

In September 2015, a 64-year-old Asian man presented to our Urology department with gradual left scrotal swelling for 2 years. His medical history included hypertension treated by orally administered Plendil (felodipine) and Acertil (perindopril), and diabetes treated by orally administered Amaryl (glimepiride) for 10 years, and hepatitis B treated by orally administered nucleos(t)ide analogs (NAs). His medical history did not include renal injury, renal biopsy, or percutaneous nephrolithotomy. He was born in Nanjing, China, and grew up in the city proper of Nanjing. His occupation is civil servant; he did not smoke tobacco or consume alcohol. No familial genetic disorder of our patient was found. On admission, his blood pressure was 141/92 mmHg, heart rate was 85 beats/minute, body temperature was 37.1 °C, and oxygen saturation was 99% on room air. A physical examination showed grade 3 left varicocele (Fig. 1a). No abnormality was found in a neurological examination. Blood chemical analyses are shown in Table 1. A urine analysis revealed urine glucose level of 2+, while other indexes including white blood cell count (3.5/ul) and red blood cell count (4.5/ul) were in the normal range. Ultrasound showed a low echo mass with fluent blood flow in the upper pole of his left kidney and bilateral varicocele (diameter, 2.7 mm for right side and 4.5 mm for left side). Ultrasound also showed a low echo of 3.7 × 4.2 cm in the upper pole of his left kidney, with affluent blood flow signals.

**Table 1:** Laboratory data on admission

Hematology	
WBC	7.42 × 10^9^/L
RBC	5.34 × 10^12^/L
Hb	161 g/L
Ht	46.8%
MCV	87.6 fL
PTL	165 × 10^9^/L
Biochemistry	
TP	72.5 g/L
ALB	50.2 g/L
T-Bil	12.1 μmol/L
γGTP	24.6 U/L
ALP	74.7 U/L
AST	18.3 U/L
ALT	28.2 U/L
LDH	197 U/L
BUN	4.39 mmol/L
Cr	85.2 μmol/L
CK	61 U/L
Na	140 mmol/L
K	3.5 mmol/L
Cl	105 mmol/L
Glu	3.62 mmol/L
CRP	3.53 mg/L

*ALB* albumin, *ALP* alkaline phosphatase, *ALT* alanine aminotransferase, *AST* aspartate aminotransferase, *BUN* blood urea nitrogen, *CK* creatine kinase, *Cl* chlorine, *Cr* creatinine, *CRP* C-reactive protein, *γ-GTP* gamma-glutamyl transpeptidase, *Glu* glucose, *Hb* hemoglobin, *Ht* hematocrit, *K* potassium, *LDH* lactate dehydrogenase, *MCV* mean corpuscular volume, *Na* sodium, *PTL* platelets, *RBC* red blood cells, *T-Bil* total bilirubin, *TP* total protein, *WBC* white blood cells

**Fig. 2:**
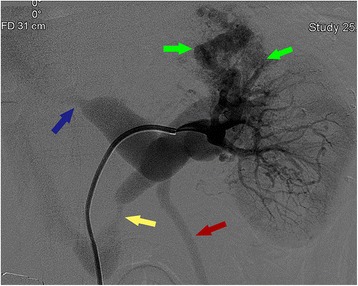
Digital subtraction angiography showed renal arteriovenous malformation by demonstrating abnormal arterial communication with vein (*green arrows*) in the upper pole with premature visualization of the dilated venous system, which included renal vein (*blue arrow*), lumbar vein (*yellow arrow*), and the left gonadal vein (*red arrow*)

**Fig. 3:**
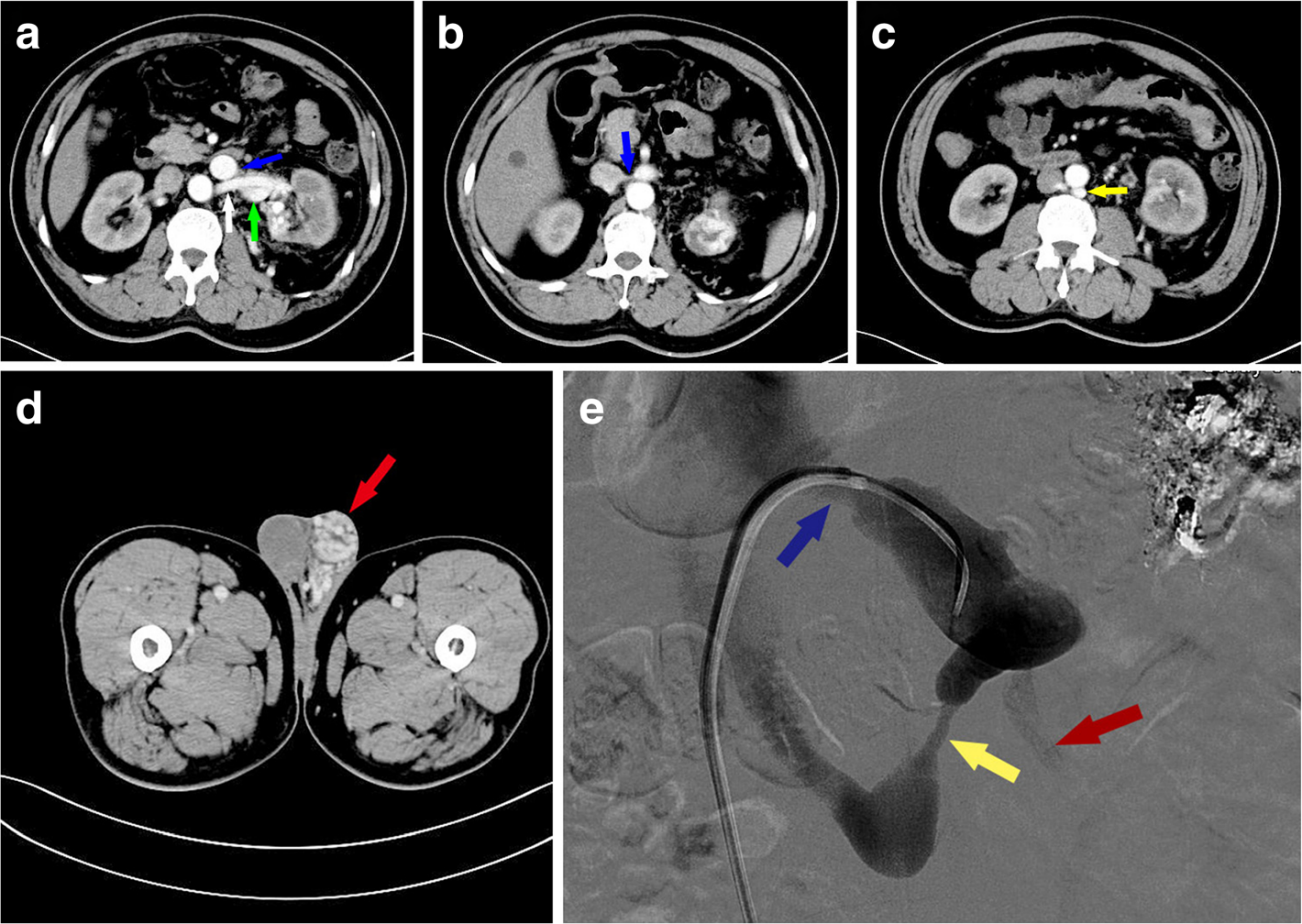
Additional radiological examination of the patient. **a** Enhanced computed tomography showed renal artery (*white arrow*), tortuous dilated vessel in left renal hilum (*green arrow*), and early enhanced renal vein (*blue arrow*). **b** Enhanced computed tomography showed early enhanced left renal vein with ostial stenosis (*blue arrow*). **c** Enhanced computed tomography showed dilated lumbar vein (*yellow arrow*). **d** Enhanced computed tomography showed enhanced left varicocele (*red arrow*). **e** Digital subtraction angiography showed lumbar vein (*yellow arrow*), light contrast in the left gonadal vein (*red arrow*) after selective embolization of the segmental renal artery, and dilation of the left renal vein ostia (*blue arrow*)

CT showed early enhanced dilated renal vein and irregular lesion in the upper pole of left kidney, which consisted of unusual dilated enhanced vessel in renal cortical phase (Fig. 1b). CT demonstrated left-side varicocele (Fig. 3d), a space-occupying lesion sized 3.6 × 4.3 cm in the upper pole of left kidney, and early enhanced dilated left renal vein with its ostial stenosis (Fig. 3b). CT also showed an unusual dilated vessel derived from left kidney and dilated lumbar vein in arterial phase, which demonstrated a local AVF (Fig. 3a and c). Axial T2-weighted MRI demonstrated large abnormal early flow voids within this mass, suggesting a vascular lesion (Fig. 1c). Selective right renal arteriography confirmed a cirsoid-type renal AVM by demonstrating abnormal arterial communication with vein in the upper pole with premature visualization of the dilated venous system, which included renal vein, lumbar vein, and his left gonadal vein (Fig. 2). Two arterial feeders arising from apical and upper segmental artery of his left kidney supply the renal AVM. He underwent selective renal artery embolization (Fig. 3e). His varicocele was alleviated obviously after selective embolization, and no relapse or abnormal blood chemical analyses were found during a follow-up time of 12 months.

## Discussion

AVFs or acquired renal AVMs are relatively rare lesions that were first described by Varela in 1928 [5]. Renal AVF usually results from trauma, biopsy, surgery, or inflammation, and the presentations of renal AVF include hematuria, hypertension, left ventricular hypertrophy, cardiac failure, abdominal pain, and flank pain [2, 6]. However, no relevant medical history or hematuria is observed in this case, although hematuria is the most common symptom of patients with renal AVF [7]. Therefore, this individual is considered an atypical case of congenital renal AVM with no hematuria. Varicocele is the primary presentation of our case. Several reported cases showed right-sided varicocele as a presentation of right renal tumor [8, 9]. An acute nontraumatic varicocele, especially on the left side, may also indicate the presence of a retroperitoneal mass [10]. In a case with varicocele, the symptom recurred after subinguinal varicocelectomy, and RCC was definitely diagnosed by histology after a radical nephrectomy [11]. However, varicocele is a rare symptom of RCC with inferior vena cava tumor thrombus, although ultrasound and CT could make the diagnosis of retroperitoneal or renal tumor in most cases [12]. Cirsoid renal AVM may be misdiagnosed as renal tumor [13, 14] or renal pelvis tumor [1] due to its remarkable similarity with renal tumor in radiological presentation. CT findings of renal AVM are commonly characterized by masses of renal sinus vascular density, which surround the pelvicaliceal system [15]. It might be very difficult to confirm the diagnosis when irregular masses in the renal parenchyma are shown by CT. A low density area in contrast-enhanced CT was suspected to indicate ischemic renal parenchyma due to the steal phenomenon of renal AVM [14]. The patient in our study presented with varicocele and renal mass, which further complicated the case. MRI may help to differentiate renal AVM from renal tumor. However, if imaged during a delayed phase after gadolinium administration, it may be difficult to differentiate an AVM from an enhancing solid renal sinus mass [16]. In this case, MRI showed a tangle of enlarged vessels and early draining vein in his left kidney, which was consistent with renal AVM [16].

Multiple arteriovenous communications, produced by a cluster of tortuous arterial and venous structures, are the main characteristics of the cirsoid-type of AVM. The preoperative diagnosis of renal cirsoid AVM is generally made with digital subtraction angiography (DSA). On angiographic examination, renal cirsoid AVM is revealed as multiple, tortuous vascular channels supplied by segmental arterial branches [17].

Angiographic embolization (AE) is being used with increasing frequency due to minimal invasion, fewer complications, and preservation of the renal function [18]. Clinically, observation is the main treatment of renal AVM in asymptomatic cases. Embolization, partial nephrectomy, and selective arterial ligation are also choices for those cases with a variety of symptoms [19, 20]. In our case, selective embolization according to previously described criteria [21] preserved most of the renal parenchyma instead of nephrectomy. Our patient has been followed up for 26 months and the regression of varicocele was observed at 6 months postoperation, and no sign of relapse of the AVM was observed.

## Conclusions

In conclusion, the etiology diagnosis of varicocele is not always straightforward, and renal AVM should be considered in the differential diagnosis of varicocele and renal mass. Renal AVM is difficult to distinguish from renal tumor according to varicocele and CT presentation, while MRI and DSA help to make a definite diagnosis and selective renal AE is one of the best treatments for renal AVM.
